# A Muscular and Cerebral Physiological Indices Assessment for Stress Measuring during Virtual Wheelchair Guidance

**DOI:** 10.3390/brainsci11020274

**Published:** 2021-02-22

**Authors:** Mohamed Moncef Ben Khelifa, Hachem A. Lamti, Vincent Hugel

**Affiliations:** 1 Impact de l’Activite Physique sur la Sante (IAPS) Laboratory, South University, 83130 Toulon-Var, France; 2 Conception de Systemes Mecaniques et Robotiques (COSMER) Laboratory, South University, 83130 Toulon-Var, France; lamtihachem@gmail.com (H.A.L.); vincent.hugel@univ-tln.fr (V.H.)

**Keywords:** BCI, EEG, EMG, virtual reality, stress, wheelchair

## Abstract

The work presented in this manuscript has the purpose to assess the relationship between human factors and physiological indices. We discuss the relationship between stress as human factor and cerebral and muscular signals as features. Ten male paraplegic, right-handed subjects were volunteers for the experiment (mean age 34 ±6). They drove a virtual wheelchair in an indoor environment. They filled five missions where, in each one, an environmental parameter was changed. Meanwhile, they were equipped with Electromyography (EMG) sensors and Electroencephalography (EEG). Frequency and temporal features were filtered and extracted. Principal component analysis (PCA), Fisher’s tests, repeated measure Anova and post hoc Tukey test (α = 0.05) were implemented for statistics. Environmental modifications are subject to induce stress, which impacts muscular and cerebral activities. While the time pressure parameter was the most influent, the transition from static to moving obstacles (avatars), tends to have a significant impact on stress levels. However, adding more moving obstacles did not show any impact. A synchronization factor was noticed between cerebral and muscular features in higher stress levels. Further examination is needed to assess EEG reliability in these situations.

## 1. Background

### 1.1. Wheelchair in Service of Disabilities

Recently, wheelchair (manual, powered) market has known a massive growth which could reach 290 million euros in 2013 [1]. France alone counts 8.1 million people affected by motor disabilities due to several pathologies (with 1.8 million use manual or powered wheelchair) where 195,268 users are in possession of manual wheelchairs. Powered wheelchairs reach roughly 10% with a market of 19,000 electric seats. Internationally, up to 650 million people (which corresponds to 10% of worldwide population) suffer from motor disabilities [2] among them 7% need a powered wheelchair. The market increase is estimated to 8% in France and 10% worldwide.

New wheelchairs integrate options and adaptive technologies to fit the user’s morphology and pathology [3,4]. While some projects focused on adapting wheelchair navigation in outdoor environments (such as [5]), others targeted mainly indoor one’s [6,7,8,9].

Ref. [10] proposed an intelligent wheelchair system based on the combination of wheelchair navigation low level algorithm’s (obstacles avoidance, path planning...) and high level techniques to set up convenient human interface interaction (tactile interaction, visual feedback...). The tests were performed on four palsy users and were divided into two phases: a training phase which consists of navigating in a virtual environment in order to familiarize with the tactile interface and an evaluation phase, where the subjects were asked to navigate through a predefined circuit (corridor following, computer room and stairs bypassing, then return to starting point). Overall assessment was based on wheelchair performance (task success, path length, time, collisions, velocity), user interface (usability, command, errors), navigation (missions, obstacles, robustness in narrow spaces) user behavior (execution, activity, competence). The results showed that subjects drove successfully the wheelchair even in the most difficult situations. Besides, the proposed system offers rehabilitation solutions for severely disabled persons.

Ref. [11] presented an extension to Montesano’s system. They incorporated an event related potentials (ERP) actuated wheelchair. Specifically, they operated with positive 300 (P300) features to select an intermediate goal point displayed on the screen. It is then transmitted to the controller which drives the electric wheelchair towards the corresponding target. Five healthy subjects took part in the experiment which includes three phases: screening and analyzing visual properties of the interface, where visual shapes, colors and placements were adapted to the user needs. Training and simulation phase was set up so that subjects familiarize with the environment and train the P300 detection algorithm. In the evaluation phase, subjects were asked to navigate through a predefined path in an indoor environment. Finally, assessments were based on the aforementioned criteria. Although subjects were able to drive their wheelchairs successfully without the need of any muscular activity, P300 was not satisfactory due to its low transfer rate and synchronization issues. They stated that some improvements are needed in this direction.

In the same context Millan et al. [12] proposed an asynchronous brain actuated control of a powered wheelchair based on motor imagery, namely event related synchronization/de-synchronization (ERD/ERS) that offers the possibility to execute different steering commands simply by modulating EEG oscillatory rhythms. The advantage of such a technique is that no external stimuli (as it is the case for ERP sources of control) are needed and commands can be issued only relying on internal activities. A shared paradigm control was implemented where a low level navigation system is activated according to the output of the high level commands issued from human interface interaction. Three subjects took part in the experiment and they underwent two steps: slalom and docking. For the former, the subjects were asked to navigate freely in the environment as the objective is to record EEG data, train the system and extract the relevant features from sensors. In the second step, docking, subjects drove mentally the wheelchair to reach target goals while avoiding obstacles. While the results seem to be encouraging as the subjects were able to control their wheelchairs (by assessing corrective actions and the percentage of reached targets), the performance is quite modest. Besides the differences between simulated and real wheelchair navigations, the delay between issued commands and wheelchair reaction is annoying and make it far from optimal.

It is true that those projects came with several enhancements to wheelchair navigation and proved its efficiencies in term of environmental awareness (obstacle avoidance, motion scheming, self localization...). However, subjects acceptability was not accounted for. From ergonomics, autonomous system’s acceptability is not taken for granted as it depends on disability level. [13] have investigated the issue with more than 110 interviews with different actors from the handicap field. They concluded (1) that users are intolerant to new features considered as a substitution of their bodies. (2) The acceptability can vary depending on the user disability (for example, Amyotrophic Lateral Sclerosis (ALS) accepts new technologies more than paraplegic). (3) Customization of new technologies is also very challenging where the introduced features must follow the disability degeneration. Such dissatisfaction promotes the appearance of negative emotions such as stress, nervousness. In our former studies [14,15,16] a comparison between healthy and disabled groups was undertaken and showed that the latter did not feel comfortable with the proposed system and we concluded that the setup of a solution to healthy people with adoption to disabled is not recommended due to acceptability differences. In this manuscript, we investigate the effect of stress on cerebral and muscular physiological indices.

### 1.2. Stress and Physiological Indices

Many projects tried to assess stress because it helps understanding of subjects- environment interaction. However, measuring and quantifying stress is very challenging. Self-report assessment are mainly used to quantify and measure stress.

#### 1.2.1. Factors Inducing Stress

Many environmental and social factors lead to stress, known as, stressors [17,18]. The types of stressors are different (sleep, deprivation, ambient temperature, noise ...) and several studies addressed:

- *adjustment to change*: When changes occur in normal routines, some levels of stress can be expressed. However proportionally to the level of change, more adjustments must be made which leads to more and more stress. Moving away from home is one of the best examples to illustrate this case: trying to fit and make new friends, adjust to the new schedule, living with strangers is very challenging and can induce stress [19]. This also depends on many factors such as the cultural and ethical backgrounds as well as geographical localization.
- *workload and overload*: It is often very difficult to decouple between stress and workload as they are strongly correlated [20]. For example, adding a workload or a secondary task to a primary one can inversely affect performance and consequently increase stress levels. In order to get overloaded, extreme or prolonged conditions of stress are required. Some projects as in [21], focused on driver’s behaviors during overload and found that their stress levels changed drastically.
- *crowding*: Many studies such as [22] demonstrated that crowding has several effects on stress, health, motivation and cognitive development. Except for family size, the density or number of people per room are relevant variables for measuring the effects of crowding [23]. The same research stated that overstimulating environments can lead to withdrawal behaviors. Extreme crowding situations are known as overcrowding.
- *Time pressure*: presented always as the most influent stressor [24], many studies addressed the impact of time pressure on cognitive performance in order to induce stress. [25] detected speech acoustic features in stressful situations. The time limit was introduced during the experiment. The experiment lasts ten minutes, but this information was hidden, until the seventh minute when subjects were informed that they should finish their mission in three minutes. A significant difference was detected between features (Pitch mean, Pitch median, Intensity max, Pitch max, Spectral tilt mean, Intensity mean, Intensity min, Intensity range, Pitch min, F1 min, Intensity std, Intensity median, F3 range) from the first seven the last three minutes. The classification rate reached 76.42%. These results suggest that deeper investigations could detect efficiently the introduction of the time pressure factor as stressor. In our previous work [26,27] time pressure was investigated as stressor as well as its influence on EEG data. It was concluded that temporal and frequency features extracted from frontal and fronto-central sensors were significantly correlated with stress levels. However, this study suggested the introduction of more stressors to assess EEG efficiency as input to detect human factors. Besides, they reported that other physiological sensors such as (EMG, ECG...) could enhance detection performance. The present work tries to deepen the latter findings by gathering another set of data and experiments.

#### 1.2.2. Assessing Stress through Physiolocal Indices

Healy et al. [28] assessed the correlation between physiological features and the emotional state of the subject while driving. The used sensors are respiration rate, skin conductance, electromyography (EMG) and electrocardiogram (ECG). Based on statistical results, predictive model based on Linear Discriminant Analysis [29] was undertaken. Although physical stress was not accounted for, they claimed that they succeeded to predict mental stress efficiently.

An extension to this study was the project held by Shi et al. [30], where physical stress was accounted for. They alternated between stressors and rest periods. Meanwhile, they collected ECG Galvanic, temperature, Skin Resistance (GSR) and respiration. Features were extracted depending on temporal duration: a frame-based which calculates features from sensors on a 60-s window. Support Vector Machine (SVM) algorithm was implemented to train the model. The precision and recall values were (0.62 ± 0.064 for frame-based and 0.68 ± 0.073 for segment-based features at 80% recall).

Ref. [31] assessed stress quantitatively using features from finger plethysmography (FPG). The tests alternates rest sessions with a period of 10 min each and Stroop color-word conflict test (CWT). Profile of Mood States (POMS) questionnaire was used for subjects feedbacks. Extracted features are high-frequency (HF) component, chaotic attractors, the largest Lyapunov exponent [32], finger pulse wave amplitude, finger pulse rate and low to-high-frequency (LF/HF) ratio. The largest Lyapunov exponent and the LF/HF ratio reported an accurate correlation with stress.

As it can be conjectured, several projects tend to find the correlation between mental stress and different types of indices either related to mental, physical or combined. Especially physiological signals of the autonomic nervous system, such as electrodermal activity and electrocardiography signals. In this way, both galvanic skin response and heart rate variability are widely used and have really good results trying to assess the arousal and valence levels of the subject. The actual study is part of a project where the main goal is to propose an intelligent wheelchair, which adapts its assistance to the subject impairment level. Consequently, the actual study addresses the following points:

- Acquire significant features from muscular activity through EMG before its degeneration and its complete loss.
- Acquire significant features from brain activity through EEG as this modality will be the only possibility for the subject to communicate with his external environment.
- Assess the correlation between both modalities in to estimate the emotional state of the subject and especially stress level.

In the current study, we simulate real situations in order to induce stress.

### 1.3. Current Study: Goals and Steps

**Figure 1:**
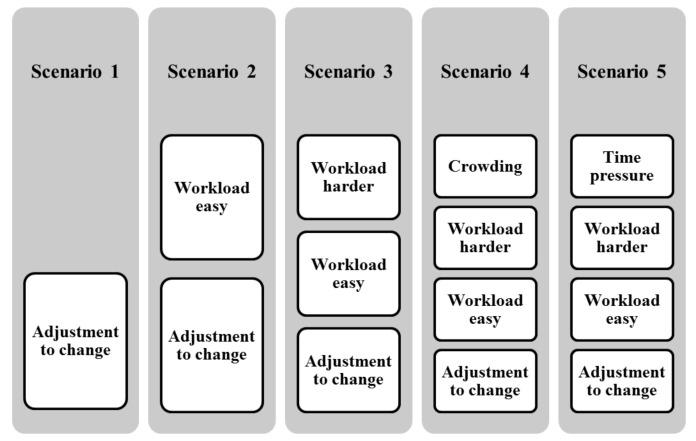
The mapping between scenarios and stressors.

A complete storyboard was implemented in the virtual reality platform. The goal is to sequentially add one of the aforementioned stressors. Five scenarios were elaborated. Each of which, simulates a situation where the specific stressor is highlighted. They are presented as follows:

- *Scenario 1: adjustment to change*: The storyboard stipulates that the subject takes the role of a newly installed roomer who must interact with his environment. He/she is guided by his roommate to visit the different rooms of the house to fit and adapt himself to the new environment.
- *Scenario 2: easy workload*: The subject is asked to collect some objects to clean the house to prepare for a night party. Some indications about the location of each object are given. The adjustment to change stressor is kept as the subject is still adapting himself to the new lifestyle, but the stress is induced through the introduced workload which is the task of collecting objects.
- *Scenario 3: harder workload*: The user has to collect the same objects placed in unknown locations with no indications given. His roommate proposes to help him finding the objects while in fact he keeps only on following the subject wherever he goes. Introducing the unknown location fact, the lack of guidance and the following avatar, increases the workload to be provided which, in turn, is assumed to increase stress level. It is very important to state that increasing the workload could lead to fatigue. However, the purpose of this experiment is to highlight stress. This specific point will be discussed later as it is difficult to decouple between fatigue and stress.
- *Scenario 4: crowding*: as the party starts, many people are coming. Here again, the subject has to gather objects from different unknown locations with no help. The introduction of many avatars in the environment makes it very cumbersome and this introduces the crowding factor as a stressor. In order to prevent the subject from providing extra maneuvers, all avatars are programmed to avoid the wheelchair at a distance of three meters (virtual environment scale). Besides, collision option is disabled, i.e., even if the avatar collides with the wheelchair, the navigation won’t be interrupted.
- *Scenario 5: Time pressure*: elapsing time was added as one of the invitees was sick and the subject has to find quickly some medicines to ensure his recovery. The medicines are placed in unknown locations and no hints are provided for guidance. The dedicated duration for this scenario is three minutes, which is fair to collect all objects. The mapping between stressors and different scenarios are summarized in the Figure 1. The goal of the current study is to assess stress by the mean of peripheral and central information features which are extracted and selected. To study correlations between scenarios and selected features, PCA, Fisher’s tests, ANOVA and adhoc Tukey *t*-tests were then used. This study addresses the following questions: What is the artifact that induces mostly the stress? which features (mental and muscular) are the most correlated with stress? is there any synchronization between mental and muscular activities over stress impact? is EEG reliable to set up systems able to detect stress during wheelchair navigations?.

## 2. Methods

### 2.1. Environment and Materials

**Figure 2:**
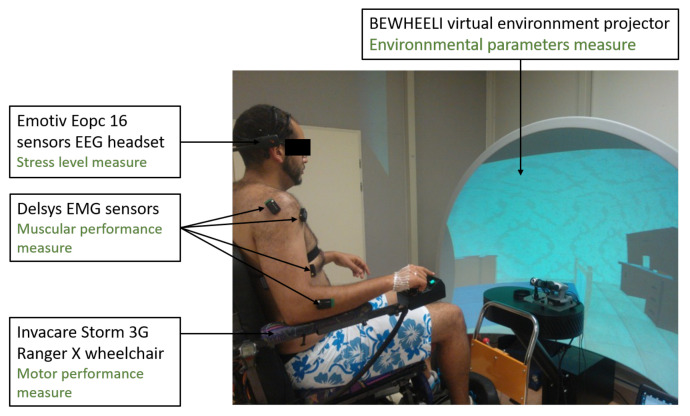
The setup of the BEWHEELI environment.

Hardware framework: A powered wheelchair from the brand (Invacare Storm 3G Ranger X) was encoded to record the velocity of the wheelchair which used to navigate in a virtual scene projected on a panoramic 180 degrees screen. A 128 Hz sampling frequency, 16 sensors Emotiv Epoc headgear is equipped to record brainwave activity. A Delsys EMG sensors down-sampled to 128 Hz sampling frequency were fixed on different locations of the right arm. In order to ensure that the sensors do not influence the subject’s valence and arousal, its choice is justified by its wireless communication as well as its non bulkiness and can be mounted easily with no need of conductive gel. The environmental setup could be found in the Figure 2.

Virtual world: The navigation scene was implemented by physics engine Reality Factory [33]. The virtual house is compound by several rooms, where artifacts are used to induce emotions and mental workload. The recourse to virtual platform is motivated by different arguments: It simulates different scenarios controlled by laboratory environment. On a real wheelchair, subjects could face crash accidents if no adequate measures are provided [34]. Moreover, as behaviors of subjects are unpredictable, this can cause accidents such as falling down.

### 2.2. Subjects

The subjects who took part in the experiment are ten right-handed (mean age 34 ±6). Their paraplegia is consequent to spinal cord injury occurring in the upper back region below the first thoracic vertebrae. Subjects benefit from full use of arms and hands, however lower limbs movements are lost. A written consent form is signed in accordance with the declaration of Helsinki. The present study was approved by the local research ethics committee in the University of Toulon (please check the joint document).

### 2.3. Scales of Emotions and Workload

**Figure 3:**
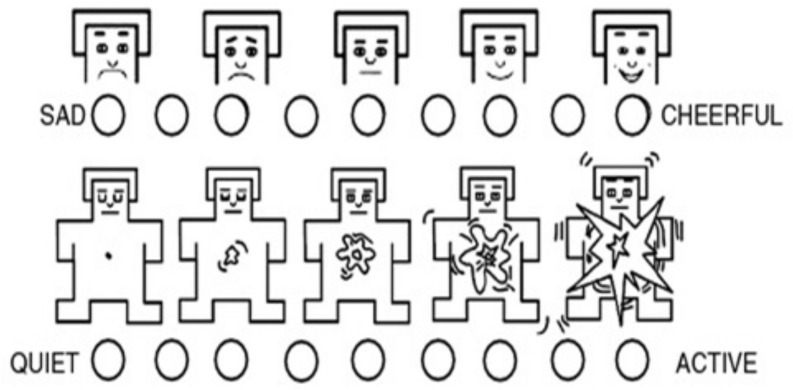
The Self Assessment Manikin (SAM) for valence-arousal assessment.

Self assessment manikin scale: As an emotional state, stress must be separated from other overlapping states (such as nervousness, anxiety...). While in some theories, emotions are presented as discrete models [35,36], others argue that emotions can be measured as differing in dimensions and degree, hence, the dimensional modeling. Pleasure-arousal [37] and approach-avoidance [38] are among the most adopted representation. In the current study, we undertake the valence-arousal representation. Every emotion is presented in a bi-dimensional space of arousal for physiological activation and valence i.e., the pleasantness within a given state. Any emotional state $e=\sqrt{v^{2}+a^{2}}$ where *v* and *a* are respectively the reported valence and arousal. Self Assessment Manikin is used rate dimensions (Figure 3).

NASA Task Load indeX scale: NASA Task Load indeX scale (NASA TLX) measures the workload in six different scales associated with different sources of workload (effort, performance, time pressure, physical demand, mental demand and frustration) [39]. The overall weighed score calculates the physical and mental workload. This measure is important to report in order to separate between stress and mental workload.

### 2.4. The Procedure of the Experiment

**Figure 4:**
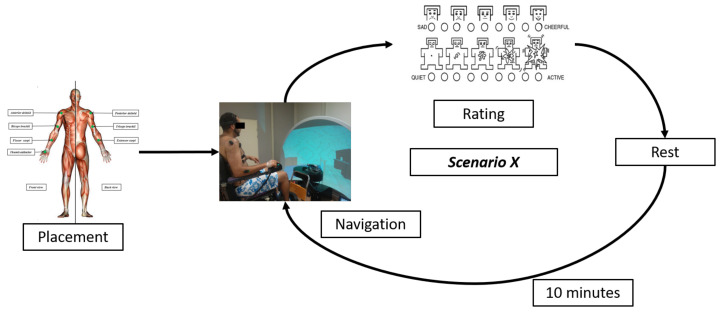
A summary of the experiment steps.

The procedure of the experiment can be summarized in the Figure 4.

#### 2.4.1. Placement of Sensors

**Figure 5:**
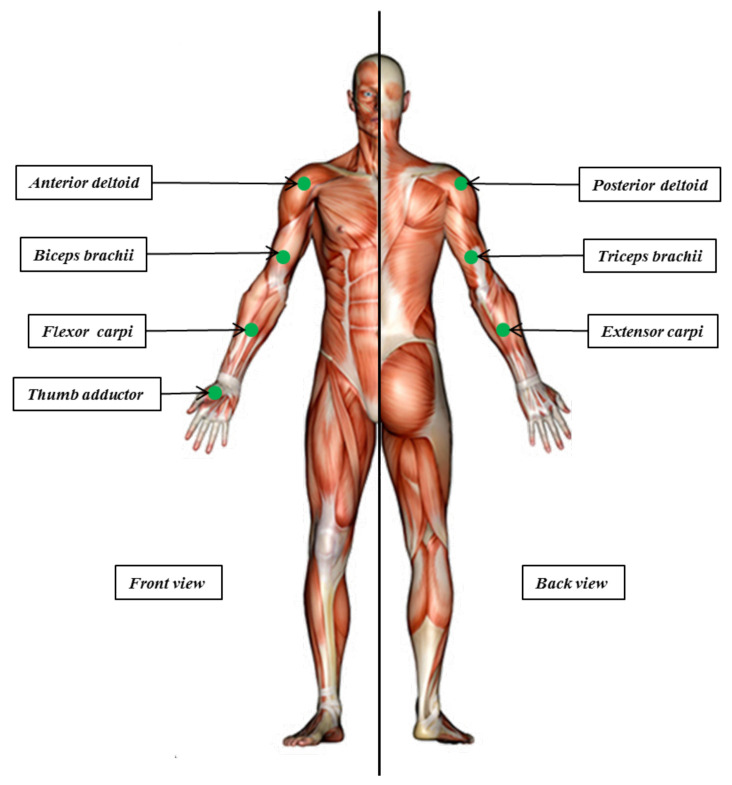
EMG sensors placement in different muscles of the right arm.

EMG sensors: After filling a consent form, EMG setup was performed. The selected locations for sensors are: the thumb adductor, biceps brachii, the wrist flexor and extensor carpi, anterior and posterior deltoid and triceps brachii (please refer to Figure 5). As a first step, and in order to get a good electrode-skin contact for better EMG recordings, subjects skins were cleaned with alcohol, shaved and rubbed with gel and abraded with sandpaper. After skin preparation, subjects were placed in a sitting posture. This starting position was adapted to determine and mark anatomical landmarks properly. Sensor location is defined as the center position of two bipolar electrodes on the muscle. This could be influenced by the presence of motor points and/or tendons as well as active muscles near sensors [40]. Next, electrodes were placed and fixed around the marked location. At this stage, inter electrode distance, orientation and fixation procedures are respected [40]. Finally Maximal Voluntary Contraction (MVC) tests were performed to check the reliability of the recorded signals and to normalize EMG features.

**Figure 6:**
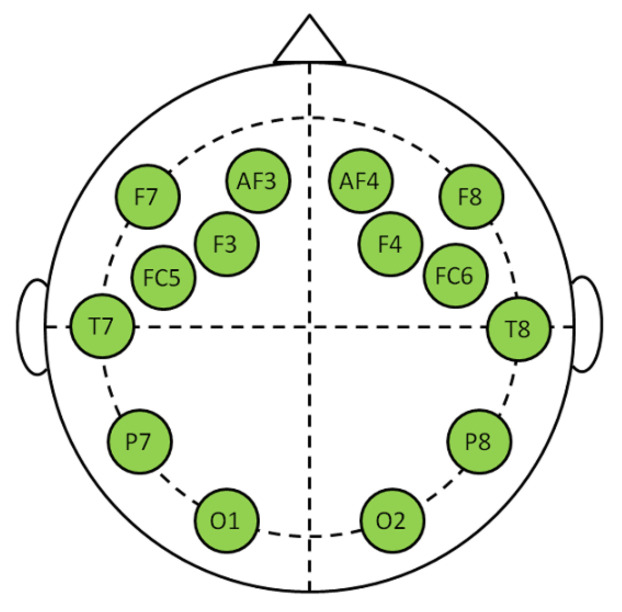
EEG sensors placed over cortex regions.

EEG sensors: Wet electrodes were arranged following the 10–20 standard [41]. Its placements are shown in Figure 6. $AF_{4},AF_{3},F_{8},F_{7},F_{4},F_{3},FC_{6},FC_{5}$ were placed in fronto-central and frontal regions. $T_{8}$ and $T_{7}$, in temporal region. $P_{8}$ and $P_{7}$ in parietal region and $O_{2}$ and $O_{1}$ in visual region. Subjects were asked to relax and close their eyes for one minute to proceed for a checking of the recorded signals.

#### 2.4.2. Procedure

After sensors placements, subjects are asked to perform a Maximal Voluntary Contraction (MVC) [42]. It is defined as the maximum force that a subject can produce during a specific isometric exercise. It is very important to account for MVC because the extracted EMG features (especially EMG activation surface) can be computed as the ratio between EMG features and the performed MVC (in this case the unit is the %MVC). To gather MVC data per muscle, subjects proceeded for several trials with 5 s each and 2 min of rest between trials. Within this duration, they rest for the first second, then generate isometric normal forces for the three following seconds than rest for the last second.

Next, subjects were asked to sit in the experimental wheelchair. The main goals of the experiment as well as how to rate emotional state were reviewed. The virtual scene consists in a house compound of a hallway and three rooms. In the bedroom, few obstacles were placed with wide inter-distances to facilitate the wheelchair navigation. In the lunchroom, more obstacles are added with narrowed space between them are extracted. The lounge is incorporated with the highest number of obstacles.

Scenarios are projected randomly following the storyboard explained earlier. In parallel with navigation, EEG and EMG features are recorded. Then, At each scenario end, subjects self-assessed the arousal, valence and workload levels by filling the NASATLX and SAM scales. Then, they rest for a period of ten minutes, assuming that this duration is sufficient to inhibit the learning effect and accumulation of stress because of scenarios succession [31].

#### 2.4.3. Features Extraction

EMG features: EMG raw data per muscle were filtered with 10th order, high-pass Butterworth filter at 20 Hz, full-wave rectified and followed by a 3rd order Butterworth low-pass filter at 5 Hz [43]. The next step consists on extracting the needed features such as the muscular activation surface which is defined as the ratio between the linear envelope of the filtered signal and the computed MVC features [44], the maximum and mean (expressed in %MVC). Power Spectral Density (PSD), the maximal, the mean, the surface and the median frequency are extracted from the frequency domain. A full list of the EMG features can be found in the Table 1.

EEG features: EEG signals were filtered with Blind Source Separation (BSS) technique [45]. Welch method was used to calculate brainwave signals [46]:

$${\hat{S}}_{f}\left(f\right)=\frac{1}{JNI}\sum_{i=0}^{J-1}|\sum_{z=0}^{N-1}{f(z)x(z+iD)exp(-jfz)|}^{2}$$

where: $I=\frac{1}{N}\sum_{i=0}^{N-1}f{\left(z\right)}^{2},N$ is the length of the window f(z), x(z+iD),i=1,2,3,...,K,K uncorrelated data of a random process x(z) over an interval 0≤z≤I. The chosen frequency intervals are between 1 Hz and 64 Hz with a window of 256 samples generating different frequency bands of δ (up to 4 Hz), θ (4 Hz–8 Hz), α (8 Hz–13 Hz) β (13 Hz–30 Hz) and γ (30 Hz–64 Hz). The standard deviation, maximum and the mean of the five frequency bands were extracted. The differences between spectral power of all symmetrical pairs of electrodes on the right and left hemisphere were computed to check if asymmetries in brain activities occurred [47]. A full detailed list of extracted EEG features can be found in Table 2.

### 2.5. Statistical Analysis

**Figure 7:**
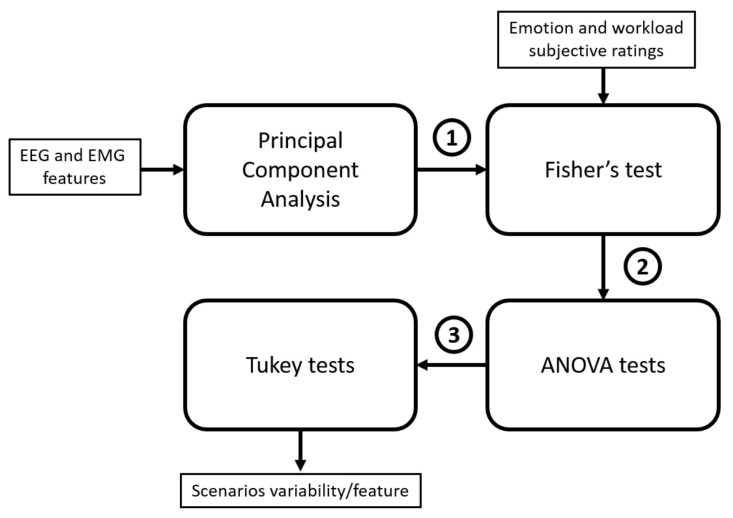
Statistics analysis methodology: presentation of the outputs of each block: 1—uncorrelated features after PCA selection. 2—Correlated features with subjective ratings. 3—Features where at least two scenarios were different.

The extracted features (7 muscles × 7 features + 21 sensors × 5 frequency band × 3 parameters) lead to the curse of high dimensionality [48]. Consequently, uncorrelated and pertinent features must be selected based on the following statistical analysis (presented in Figure 7):

- Initially, independence between features was undertaken using PCA. Following the method presented by Rocchi et al. [49] to select relevant features which are used as input for the correlation block.
- In order to assess the correlation between EEG, EMG selected components and subjective ratings, Fisher’s method [50] is apprehended. *p*-values and spearman correlated coefficients were calculated between features and ratings for each participant. Assuming independence [51], Fisher’s method is used to combine the resulting *p*-values into one *p*-value. Finally, features with significant correlations (p<0.05) were selected.
- ANOVA and Tukey tests were performed to assess the variability between the effect of stress level on features and the scenarios. The reported scores will give us an idea about the efficiency of the artifacts to reach the needed level of stress and its impact on physiological sensors.

#### 2.5.1. Principal Component Analysis (PCA)

PCA procedure was applied as a feature selection tool. It transforms a set of correlated features into smaller numbers by the mean of Principal Components (PC). In our context, the purpose of PCA is to extract uncorrelated features for each scenario. The number of PCs to retain was calculated based on Kaiser criterion [52] (keeping PCs whose eigenvalues are greater than one). Besides, in order to limit the chosen PCs, an investigation was carried out to calculate the suitable threshold which ensures that all retained PCs have consistent weights and hold acceptable percentage to explain original features. For example, if the threshold is fixed at 92% and the cumulated sum between the first (60%) and second (30%) PCs is 90%, the third PC (2%) is not very consistent. Consequently, after tests, 89% was chosen as the best trade-off between PCs weights and explained features.

The selection process described by Rocchi et al. [49] was adopted and summarized in the following steps:

- Compute PCs for each scenario separately and for all scenarios. While computing PCs for all scenarios is used to extract uncorrelated features coarsely, computing PCs for each scenario validates the main features already found in all scenarios and accounts for small variations between scenarios. Consequently, more detailed features can be detected.
- Calculate the new weights of the features in the PC.
- Calculate the correlation coefficient between features and PC based on the following equation:
  $$r_{ij}=\sqrt{\frac{a_{ij}^{2}Var\left(Y_{j}\right)}{s_{ii}}}$$
  where $r_{ij}$ is the correlation coefficient between the feature $X_{i}$ and the principal component $Y_{j}$. $a_{ij}$ is the weight of the the feature $X_{i}$ in the principal component $Y_{j}$ which correspond to the eigenvectors of the variance-covariance matrix S. $s_{ii}$ are the eigenvalues of the matrix S. They represent the variance explained by each PC.
- Attribute an occurrence index to each highly correlated feature per PC and per scenario. This index will reveal if the corresponding feature is redundant in scenarios (and in this case it is relevant to be considered for the Fisher’s tests).
- Finally, dress a ranking list with features, their correlation coefficients and their occurrence indices. Features with the highest occurrence score are retained.

#### 2.5.2. Spearman Coefficients and Fisher’s Test

Although selected features from PCA block are uncorrelated, appropriate correlations with stress levels is not evident: even features with smaller variations in the selected PCs, can be better related to stress. To this end, the correlation between subjective ratings and selected features are investigated. For each subject, the input matrix M gathers the different features issued from the PCA selection phase. State matrix S contains all subjective ratings (emotion and workload) where emotion *e* is computed as $e=\sqrt{a^{2}+v^{2}}$ [47] where *a* is the arousal and *v* the valence of the corresponding scenario. M and S are initialized as follows:

(1) $$M=\left(\begin{array}{ccc} m_{1,1} & \ldots & m_{1,length_{PCA}}\\ \vdots & \ddots & \vdots \\ m_{5,1} & \ldots & m_{5,length_{PCA}} \end{array}\right)$$

(2) $$S=\left(\begin{array}{cc} s_{1,1} & s_{1,2}\\ \vdots & \vdots \\ s_{5,1} & s_{5,2} \end{array}\right)$$

where: $m_{i,j}$ is the measure associated with the scenario *i* and feature *j* retained from the PCA selection phase. $length_{PCA}$ denotes the total number of uncorrelated features suggested by the PCA block and $s_{i,j}$ is the subjective rating (emotion and workload) reported by the subject in the *i*th scenario. Spearman correlated coefficients were computed between features and the subjective ratings, as well as the *p*-values, (*p*). The spearman coefficient is calculated as follows:

(3) $$p=1-\frac{6\sum d_{i}^{2}}{n(n^{2}-1)}$$

where: $d_{i}$ is defined as $d_{i}=x_{i}-y_{i}$ in each observation, $x_{i}$ and $y_{i}$ are the ranks of the raw scores $X_{i}=m_{i,j}$ and $Y_{i}=s_{i,j}$ and *n* is the number of samples. This was performed for each subject individually and, assuming independence, the resulting *p*-values per feature were then combined to one *p*-value via Fisher’s method:

(4) $$\chi^{2}=-2\sum_{i=1}^{k}log_{e}\left(p_{i}\right)$$

where: $p_{i}$ is the *p*-value associated to the subject *i* and k=10 is the total number of subjects in this experiment.

#### 2.5.3. Anova and Tukey Tests

Once features were selected, ANalysis Of VAriance (ANOVA) and Tukey HSD tests were set up to assess the effect of stress on features and study the inter-scenarios variability. In order to apply Tukey tests, the measurements within and among groups must be uncorrelated. In fact, as users go sequentially through all scenarios (and not in a randomized order), a learning effect can occur. We assume that the ten minutes of inter-scenarios rest, are sufficient to inhibit this effect and ensure independence between conditions. Tukey test can accurately maintain alpha levels at their intended values provided that some assumptions are made on the model (normality, independence and homogeneity...). The tukey formula can be expressed as follows:

$$HSD=q\sqrt{\frac{MSE}{n^{*}}}$$

where *q* is the critical value of the studentized statistic for an alpha of 0.05, $n^{*}$ is the number of scores used to calculate the group means and MSE the mean square error.

## 3. Results

### 3.1. Analysis of Subjective Rating

Stress is basically a state characterized by positive arousal and negative valence [37]. The valence rate belongs to the sets: arousal rate in High={7,8,9} and valence in Low={1,2,3}. The combined load score was mapped into three different sets: Low={1,2,3}, Medium={4,5,6} and High={7,8,9}. When High set is reached, a fatigue effect is plausible to occur. Table 3 reports the standard deviation of arousal, valence and workload ratings from subjects for each experienced scenario. Initially, Valence started at (6.85) High level with a standard deviation of 3.13. This variability between subjects is proportional to the first impression reported by subjects: some subjects rated very high as they assimilate it to video games while others rated it very low. However, as they enchain scenarios, both the rating mean and the standard deviation decrease where at the fourth and fifth scenarios, the mean ratings reached the Low set with 2.85 (1.21) and 3 (1.5) respectively. Inversely, arousal ratings changes from 5.14 (3.53) in the first scenario to 7.28 (1.88) in the fifth scenario. The distribution of ratings suggests a transition from relaxed to stressed state as mentioned in [47,53]. Consequently, the emotional state will be referred to as stress.

The combined workload score increases linearly from the first scenario 2.3 (1.78) to the fourth scenario 3.7 (1.3) with a difference of 1.4. However, this difference increases to reach 1.5 only between the fourth and fifth scenario reaching 5.2 (0.96). This means that time pressure stressor is workload consuming. However, this measure is still in the Medium range. This could be explained by the fact that the duration of the elapsing time is for three minutes, which is not enough to induce a higher level of physical and cognitive workloads which can lead to fatigue.

### 3.2. Correlation between Stress and Emg Features

**Figure 8:**
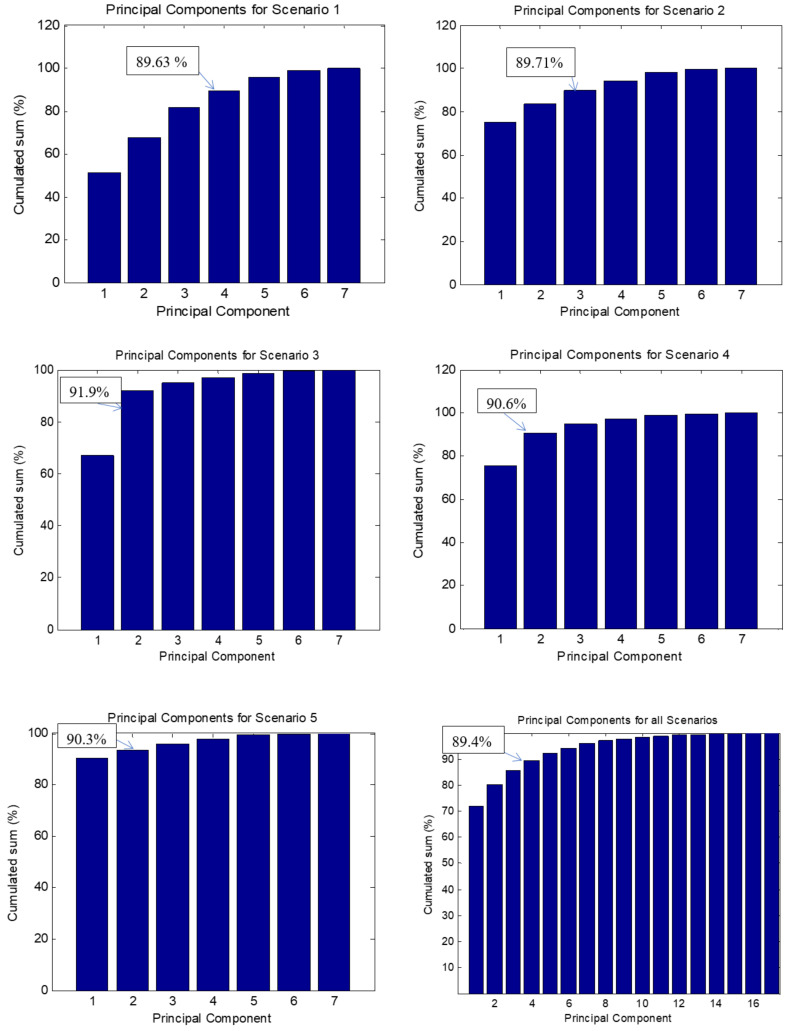
From left to right: the cumulated sum associated to principal components for each scenario (1 to 5) and for overall scenarios. Only PCs with cumulated sum greater than 89% are kept.

PCA and Fisher’s tests were used to select significant EMG and EEG features with regard to stress levels. Figure 8 illustrates the PCs kept for each scenario whose cumulated sum are over 89%. The number of PCs is different from scenario to another: while in the first scenario 4 PCs were preserved, this number started to decrease (3 for the second, 2 for the third and fourth scenarios and only one is sufficient for the fifth scenario). Table 4, Table 5, Table 6, Table 7 and Table 8 report the coefficients and the correlation indices of the most significant features (where correlation |r|>0.4 [49]). By attributing an occurrence index to each selected feature with accounting for the correlation coefficients, a ranking can be listed. Using Fisher’s tests, *p*-values were computed for each selected feature and only significant correlations (p<0.05) with stress were reported. The most correlated features are $Thumb_{Max}^{Amplitude}$ ($p_{stress}=0.0014$, $p_{workload}=0.08$), $Thumb_{Surface}^{EMG}$ ($p_{stress}=0.024$, $p_{workload}=0.098$) and $Extensor_{Surface}^{EMG}$ ($p_{stress}=$0.049, $p_{workload}=0.1$). Notice that these features were not correlated with workload ratings. This is linked with the previously reported results: workload did not reach a certain level where fatigue can occur and supports the assumption that the inferred results are related to stress, but not mental workload.

### 3.3. Correlation between Stress and Eeg Features

**Figure 9:**
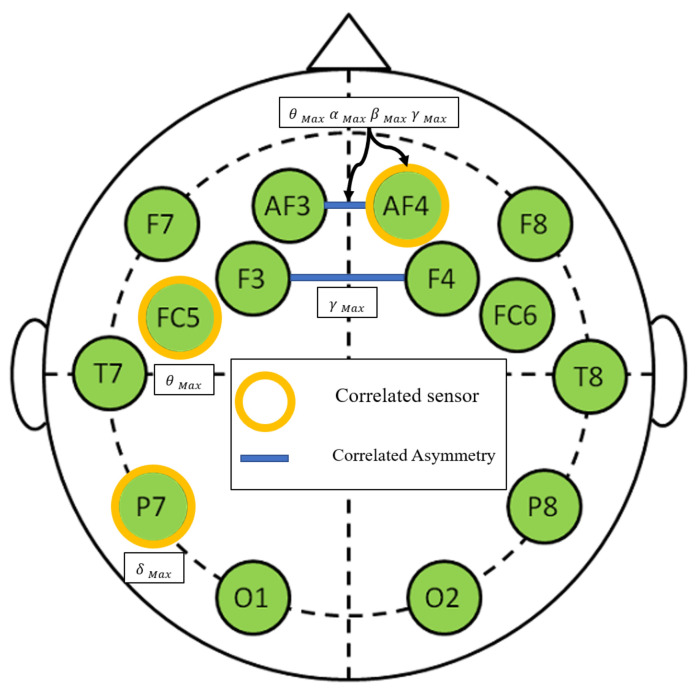
EEG correlation illustrated by band-wave, sensor, region and feature.

Following the same process to select EMG features using PCA and Fisher’s tests, it has been found that $FC5_{Max}^{\theta }$ ($p_{stress}=0.0010$, $p_{workload}=0.062$), $P7_{Max}^{\delta }$ ($p_{stress}=0.0011$, $p_{workload}=0.065$), $AF4_{Max}^{\theta }$ ($p_{stress}=0.002$, $p_{workload}=0.07$), $AF4_{Max}^{\alpha }$ ($p_{stress}=0.0022$, $p_{workload}=0.054$), $AF4_{Max}^{\beta }$ ($p_{stress}=0.0035$, $p_{workload}=$0.085), $AF4_{Max}^{\gamma }$ ($p_{stress}=0.003$, $p_{workload}=\;0.085$) reported the best correlation rate. Consequently, parietal regions, maximum amplitude of different band-waves in fronto-central and frontal are the most influenced. Besides, ${\left(AF4-AF3\right)}_{Max}^{\theta }$ ($p_{stress}=0.0009$, $p_{workload}=0.055$), ${\left(AF4-AF3\right)}_{Max}^{\alpha }$ ($p_{stress}=0.0010$, $p_{workload}=0.059$),

${\left(AF4-AF3\right)}_{Max}^{\beta }$ ($p_{stress}=0.0014$, $p_{workload}=0.065$), ${\left(AF4-AF3\right)}_{Max}^{\gamma }$ ($p_{stress}=0.0018$, $p_{workload}=0.071$), ${\left(F4-F3\right)}_{Max}^{\gamma }$ ($p_{stress}=0.002$, $p_{workload}=\;$0.075). We notice a better correlation between EEG features and workload ratings (although they are >0.05). This means that EEG is more sensitive to workload variations than EMG. This means also that EEG is a very good candidate to be considered for mental workload than EMG. Those results report a high correlation between the asymmetries for different band-waves in frontal region, especially for AF4 and AF3 and stress levels. Figure 9 illustrates correlations per feature, band-wave and region asymmetries.

### 3.4. Effects of Stress on Different Scenarios

#### 3.4.1. Anova Tests

Repeated measure ANOVA was applied on EMG and EEG selected features. As it is shown in Table 9 and Table 10, significant effects were reported for features $Thumb_{Max}^{Amplitude}$, $Thumb_{Surface}^{EMG}$ and $Extensor_{Surface}^{EMG}$ with highest correlation associated with the first feature (*F*(4,49) = 3.89, *p*-value = 0.0124) and lowest with the second (*F*(4,49) = 2.89, *p*-value = 0.0403). The same observation was found for EEG selected features. Strong correlations were reported for $AF4_{Max}^{\theta }$, $AF4_{Max}^{\alpha }$, ${\left(AF4-AF3\right)}_{Max}^{\theta }$, ${\left(AF4-AF3\right)}_{Max}^{\alpha }$ with respectively (*F*(4,49) = 5.57, *p*-value = 0.00198), (*F*(4,49) = 4.43, *p*-value = 0.006), (*F*(4,49) = 7.36, *p*-value = 0.003) and (*F*(4,49) = 6.26, *p*-value = 0.001).

#### 3.4.2. Posthoc Tukey Tests

**Figure 10:**
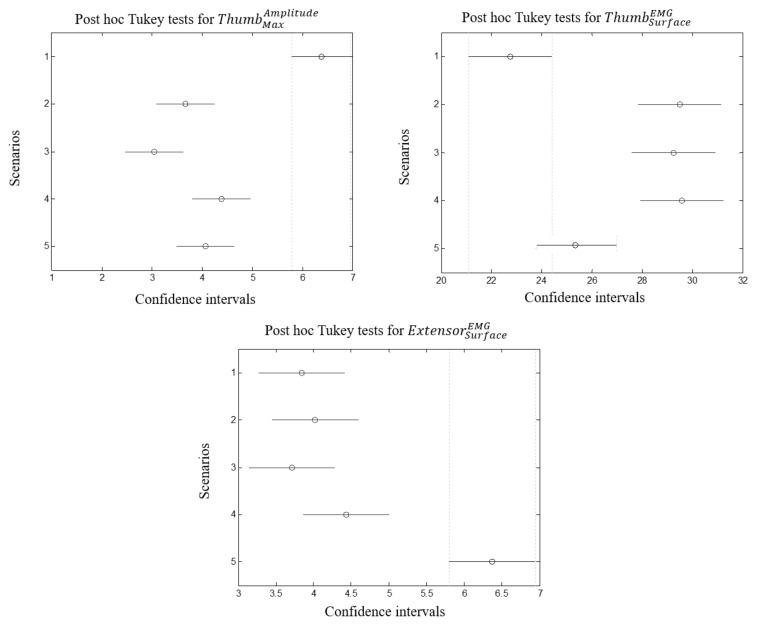
From left to right: differences between scenarios reported by Tukey tests for $Thumb_{Max}^{Amplitude}$, $Thumb_{Surface}^{EMG}$ and $Extensor_{Surface}^{EMG}$.

**Figure 11:**
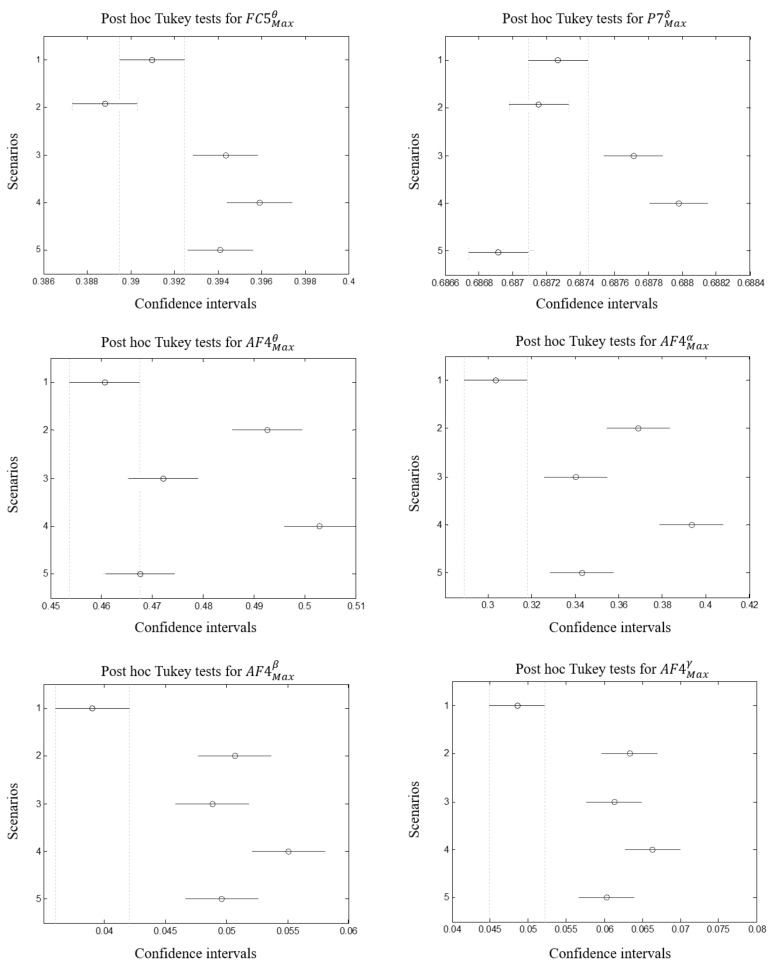
From left to right: differences between scenarios reported by Tukey tests for $FC5_{Max}^{\theta }$, $P7_{Max}^{\delta }$, $AF4_{Max}^{\theta }$, $AF4_{Max}^{\alpha }$, $AF4_{Max}^{\beta }$, $AF4_{Max}^{\gamma }$.

**Figure 12:**
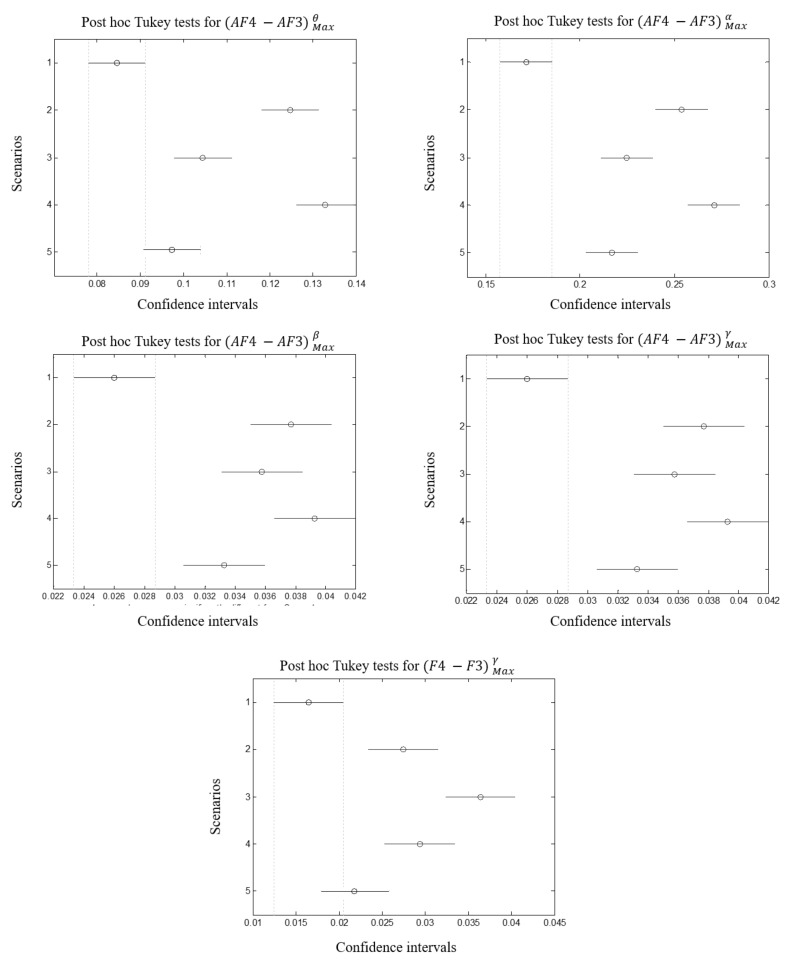
From left to right: differences between scenarios reported by Tukey tests for $FC5_{Max}^{\theta }$, $P7_{Max}^{\delta }$, $AF4_{Max}^{\theta }$, $AF4_{Max}^{\alpha }$, $AF4_{Max}^{\beta }$, $AF4_{Max}^{\gamma }$.

Differences between scenarios are assessed regarding the selected features, posthoc tukey tests were performed with α = 0.05. Figure 10 reports the mean differences between groups for each selected EMG feature whereas Figure 11 and Figure 12 report those for EEG selected features per sensor and per brain asymmetries. The overall tests reveal that the first scenario showed difference from the others (although it was not the case for $Extensor_{Surface}^{EMG}$). In some cases the latter is overlapping with the second or the fifth scenarios. Tukey tests on EMG features reveal that the second, third and fourth scenarios did not show significant differences while the fifth showed difference for $Extensor_{Surface}^{EMG}$ and $Thumb_{Surface}^{EMG}$ but not for $Extensor_{Max}^{Amplitude}$. On the other hand, Tukey tests on EEG features reveal that the first scenario showed differences with at least two scenarios especially third and fourth. The second and third scenarios were different in several cases. Third and fourth scenarios did not show differences in most cases. The fifth scenario was different from the second, third and fourth scenarios. Theses differences are more evident with brain asymmetries especially with the difference between the fifth scenario and the second, third and fourth scenarios although it is not always the case.

## 4. Discussion

### 4.1. Effects of Environmental Changes on Stress

It can be conjectured from the results that environmental changes between scenarios had more or less effect on stress level. In fact, the first scenario was easily differentiated from the others: each added artifact had its influence to induce stress. This means that either from muscular or cerebral activity or both, conceiving a system to predict stress on the subjects is possible. The second, the third and the fourth scenarios (where object locations passed from known to unknown and from static to moving obstacles) did not show many differences. It can be explained by the fact that in the second scenario, the subjects faced for the first time a stressful situation then they became more and more familiar with them. Besides, modifying an environmental parameter (such as number of obstacles, velocity ...) did not impact the stress level. However, introducing a new artifact such as time pressure had more impact on the user’s state. Also, the presentation sequence can have an influence on stress manifestation: while in this study a logical series was followed as to put subjects in a real situation, alternating between restful and stressful scenarios could help to study each parameter individually and not in comparison with others.

### 4.2. Muscular Activity Compensation

From all attached EMG sensors, thumb location was significantly correlated with stress levels. It is plausible that muscular activity could be explained by the effort provided by the subject to avoid obstacles. However, this issue was accounted for when programming the virtual environment (example, if the wheelchair is in the range of a moving avatar, the latter manages to avoid collision). The first scenario showed difference from the others especially for $Thumb_{Surface}^{EMG}$. The fifth scenario was significantly different for $Extensor_{Max}^{Amplitude}$ feature. Assuming that time pressure is the stressor artifact, it can be stated that extensor is activated at higher levels of stress. For the second, third and fourth scenarios no differences were shown. This can be interpreted by the fact that even if the subject is stressed, he managed to navigate and finish the missions without impact on EMG activity. Another interpretation suggests that the introduced artifact within each new scenario, except time pressure, did not bring evident changes. This also suggests that scenario 2, 3 and 4 must be fused into one scenario. In this case, differences could be more visible. However, Those conjectures are only valid for paraplegic group, another group which suffers from severer disability could lead to other conclusions.

### 4.3. Cerebral Activity Changes

Fronto-central, parietal and frontal regions of the brain cortex correlate with stress levels where all bandwaves from AF4 sensor gave significant results. Consequently, only AF4 sensor is sufficient to predict stress levels. However, the differences are less evident even if EEG results were more explicit than EMG. In fact, scenarios 2, 3 and 4 show evident differences especially for $AF4_{Max}^{\alpha }$ and $AF4_{Max}^{\theta }$. Brain asymmetries report that γ and θ,α over (AF4-AF3) are correlated with stress. [47] reported the same findings where θ and α increase were correlated with emotional levels. While the fifth scenario can be differentiated from the others, scenarios 2,3 and 4 showed the least differences. This confirms that the modifications between these scenarios are not sufficient to induce a higher level of stress which is not the case for the time pressure. This can be interpreted by the fact that switching from non to stressful scenario (scenario 1 to 2) impacts the behavior of the subject. The latter is compensated when passing from scenario 2, 3 to 4 as the subject became more familiar with the environment and masters the wheelchair driving. The similitudes between those scenarios made the level of stress less evident where the objects, the obstacles, the avatars were repeated. However in the fifth scenario, objects, elapsing time and context favored the appearance of the stress level.

### 4.4. De-Synchronization between Brain and Muscles

By comparing the results of EEG with those of EMG, the former showed more correlation with stress level (especially for the first and fifth scenarios) while in the latter, only the first scenario was different from the others. This means that even though the subject was stressed, he managed to drive successfully his wheelchair over the environment thanks to EMG compensations. We label this as de-synchronization between brain and muscles. In the last scenario, where the stress level is the highest, EEG and EMG activations were synchronized. Consequently, and in order to have an evident synchronization with EEG and EMG, high levels of stress must be induced. In real world, stressful situations can vary drastically from low to high level in few seconds. This synchronization factor can be very useful to differentiate between low and very high stress levels. However, to differentiate between low and normal or normal-to-high stress levels, EEG activity can be sufficient.

### 4.5. Reliability of EEG

Although EEG features were sensitive enough and can be used as inputs of a predictive model for stress levels, the validation of this fact cannot be confirmed due to many problems: EEG was not efficiently sensitive to detect small changes in stress levels: features correlations were relevant only between first and the set compound with the second, third and fourth scenarios or between the latter and the fifth scenario. However, within the same set, the differences are less evident. Enlarging datasets with more samples could resolve this problem. Moreover, the results could be enhanced by including subjects with more severe disabilities: many studies showed that environment artifacts can influence the results [54]. It is recommended to use the EEG jointly with EMG features to detect higher stress levels and to exploit the synchronization factor which occurs between them. In future projects, different physiological sensors will be added to assess and detect stress. In fact, the only use of EEG is not efficient. However, the context of the actual project imposes the use of brain activity to command and control wheelchairs, the use of EEG will be maintained in addition to other physiological sensors that could enhance stress detection. In this case, the challenge is to assess the trade-off between robustness of the detection and the minimum set of combined physiological sensors activities (example {EEG,HRV}, {EEG,EOG}, {EEG,EMG,HRV}...).

### 4.6. Shortages of the Current Findings

This experiment was based on several assumptions which can impact the efficiency of the findings. First, the study is based on correlation between physiological sensors and subjective ratings (emotion and mental workload). Although this technique is widely adopted in several projects like [28,47] the subjectivity of the ratings limits all findings as the differences between subjects are sensitive to mistaken the reported measures. In other words, measuring and quantifying mental states is very challenging. Consequently, statistics and results which are based on the direct relation between stress, scenarios and physiological measures could be biased. In fact, other factors can overlap with stress, such as mental workload (even in subjective ratings this latter is limited) or other mental events (such as developing mental strategies to solve tasks or concentration while searching in the environment...). Those are linked to the complexity of the brain. Second, the assumption of ten minutes rest between scenarios is also to be discussed. In fact, the trade-off between stress induction and learning effects is still difficult to find. Also, this assumption could be insufficient to inhibit learning effect and though acquire the needed stress effect to be studied afterwards.

## 5. Conclusions

The impact of stress on muscular and cerebral was assessed through EMG and EEG. To this end, navigation scenarios were created where, in each one, an environmental artifact was embedded to induce stress. Thumb muscle was highly correlated with stress levels. On the other hand, for EEG features, parietal and frontal regions were the most correlated. However, the results are different from non-stressful to stressful scenarios and between the introduced artifacts where time pressure tends to show the highest impact. A de-synchronization factor was observed between EEG and EMG in small changes between scenarios, but becomes more evident in higher stressing situations. On the other hand, many interesting points are subject to deeper investigations especially EEG reliability. Besides, the EMG in this study is down-sampled to a rate of 128 Hz and limited to the envelope of the signal. Since the information of the EMG is presented in a wider range of frequencies, the evaluation of higher frequencies of the signal could be very interesting to end up with further conclusions. The correlations between subjective ratings and physiological sensor recordings is also questionable as the subjectivity in mental state is still a trendy issue. The next step will consist of building a model for intelligent techniques such as SVM, neural networks... to predict stress levels. Another perspective is to deal with the relationship between stress, mental fatigue and EEG features.

## 6. Ethical Approval

All procedures performed in studies involving human participants were in accordance with the ethical standards of the institutional and/or national research committee and with the 1964 Helsinki declaration and its later amendments or comparable ethical standards.

## 7. Key Points

(1) *EMG features correlations*: From EMG selected features, the thumb muscle tends to be the most influenced by stress inductions.

(2) *EEG features correlations*: Many brain regions showed correlation with respect to stress induction experiment such as frontal, fronto-central and parietal regions. Besides many asymmetries were correlated.

(3) *De-synchronization between EMG and EEG*: A synchronization factor was noticed between EEG and EMG only on high stress levels which was previously compensated when stress level was lower

(4) *Effects of environmental changes on stress induction*: Time pressure tends to be the most influencing stressor while the introduction of moving avatars did not affect subjects.

## Figures and Tables

**Table 1:** Features extracted from EMG.

Muscle	Extracted Features/Muscle	Definition
Thumb adductor	$Amplitude_{Max}$	(in %) percentage of the maximum peak with respect to the MVC
Flexor carpi	$Amplitude_{Mean}$	(in %) percentage of the mean amplitude with respect to the MVC
Extensor carpi	$EMG_{Surface}$	(in %) percentage of the EMG surface with respect to the MVC
Biceps brachii	$PSD_{Max}$	(in db) The maximum power spectral density
Triceps brachii	$PSD_{Mean}$	(in db) The mean power spectral density
Anterior deltoid	$Frequency_{Surface}$	(in db/Hz) The surface of the power spectral density
Posterior deltoid	$Median_{Frequency}$	(in Hz) The medium frequency

**Table 2:** Features extracted from EEG.

Sensor	Extracted Features/Sensor	Definition
AF3	$\delta_{Max}$	Maximum Delta power
F7	$\delta_{Mean}$	Mean Delta power
F3	$\delta_{Std}$	Standard deviation Delta power
FC5	$\theta_{Max}$	Maximum Theta power
T7	$\theta_{Mean}$	Mean Theta power
P7	$\theta_{Std}$	Standard deviation Theta power
O1	$\alpha_{Max}$	Maximum Alpha power
O2	$\alpha_{Mean}$	Mean Alpha power
P8	$\alpha_{Std}$	Standard deviation Alpha power
T8	$\beta_{Max}$	Maximum Beta power
FC6	$\beta_{Mean}$	Mean Beta power
F4	$\beta_{Std}$	Standard deviation Beta power
F8	$\gamma_{Max}$	Maximum Gamma power
AF4	$\gamma_{Mean}$	Mean Gamma power
AF4-AF3	$\gamma_{Std}$	Standard deviation Gamma power
F8-F7		
F4-F3		
FC6-FC5		
T8-T7		
P8-P7		
O2-O1		

**Table 3:** Mean(standard deviation) of valence, arousal and workload ratings for different scenarios.

Scenario	Sc1	Sc2	Sc3	Sc4	Sc5
Valence	6.85 (3.13)	6.14 (2.6)	4.57 (2.3)	3 (1.15)	2.85 (1.21)
Arousal	5.14 (3.53)	5.42 (2.14)	6.14 (1.46)	6.85 (0.89)	7.28 (1.88)
Workload	2.3 (1.78)	2.5 (0.85)	2.72 (1.3)	3.7 (1.3)	5.2 (0.96)

**Table 4:** PC coefficients and correlations between features and corresponding PC for the first scenario. Only |r|>0.4 are shown.

	PC1				PC2		
	51.5%				16.4%		
**Muscle**	**Feature**	**Coefficient**	**Correlation**	**Muscle**	**Feature**	**Coefficient**	**Correlation**
Thumb	$Amplitude_{Max}$	0.37	0.89	Thumb	$Amplitude_{Mean}$	0.69	0.81
Thumb	$EMG_{Surface}$	0.67	0.96	Thumb	$PSD_{Mean}$	0.05	0.55
Extensor	$Amplitude_{Max}$	0.065	0.75	Thumb	$Median_{Freq}$	−0.021	−0.49
Extensor	$EMG_{Surface}$	0.19	0.87	Extensor	$Amplitude_{Mean}$	0.2	0.59
Flexor	$Amplitude_{Max}$	0.14	0.84	Flexor	$Surface_{Freq}$	0.25	0.56
Flexor	$EMG_{Surface}$	0.48	0.86				
	**PC3**				**PC4**		
	**14%**				**7.83%**		
**Muscle**	**Feature**	**Coefficient**	**Correlation**	**Muscle**	**Feature**	**Coefficient**	**Correlation**
Extensor	$Amplitude_{Mean}$	0.12	0.54	Thumb	$PSD_{Mean}$	−0.081	−0.61
Extensor	$EMG_{Surface}$	−0.18	−0.42	Thumb	$Median_{Freq}$	−0.029	−0.45
Flexor	$Amplitude_{Mean}$	0.27	0.56	Extensor	$PSD_{Mean}$	−0.031	−0.85

**Table 5:** PC coefficients and correlations between features and corresponding PC for the second scenario. Only |r|>0.4 are shown.

	PC1				PC2		
	74.9%				8.55%		
**Muscle**	**Feature**	**Coefficient**	**Correlation**	**Muscle**	**Feature**	**Coefficient**	**Correlation**
Thumb	$Amplitude_{Max}$	0.27	0.87	Thumb	$Amplitude_{Mean}$	0.49	0.57
Thumb	$Amplitude_{Mean}$	0.19	0.66	Thumb	$PSD_{Mean}$	0.086	0.5
Thumb	$EMG_{Surface}$	0.64	0.98	Thumb	$Median_{Freq}$	0.11	0.82
Extensor	$EMG_{Surface}$	0.236	0.97				
Flexor	$Amplitude_{Max}$	0.127	0.85				
Flexor	$EMG_{Surface}$	0.0047	0.97				
	**PC3**						
	**6.26%**						
**Muscle**	**Feature**	**Coefficient**	**Correlation**				
Thumb	$Amplitude_{Max}$	0.46	0.43				
Thumb	$Amplitude_{Mean}$	−0.42	−0.42				
Thumb	$Surface_{Freq}$	0.0573	0.42				

**Table 6:** PC coefficients and correlations between features and corresponding PC for the third scenario. Only |r|>0.4 are shown.

	PC1				PC2		
	67.1%				24.8%		
**Muscle**	**Feature**	**Coefficient**	**Correlation**	**Muscle**	**Feature**	**Coefficient**	**Correlation**
Thumb	$Amplitude_{Max}$	0.11	0.63	Thumb	$Amplitude_{Mean}$	0.95	0.99
Thumb	$EMG_{Surface}$	0.66	0.9	Thumb	$PSD_{Mean}$	0.075	0.79
Extensor	$Amplitude_{Max}$	0.022	0.61	Extensor	$Amplitude_{Mean}$	0.042	0.43
Extensor	$EMG_{Surface}$	0.26	0.98	Extensor	$PSD_{Mean}$	0.033	0.93
Flexor	$Amplitude_{Max}$	0.051	0.67	Flexor	$PSD_{Mean}$	0.06	0.93
Flexor	$EMG_{Surface}$	0.66	0.99				

**Table 7:** PC coefficients and correlations between features and corresponding PC for the fourth scenario. Only |r|>0.4 are shown.

	PC1				PC2		
	75.4%				15.2%		
Muscle	Feature	Coefficient	Correlation	Muscle	Feature	Coefficient	Correlation
Thumb	$Amplitude_{Max}$	0.134	0.67	Thumb	$PSD_{Max}$	−0.045	−0.46
Thumb	$EMG_{Surface}$	0.62	0.98	Thumb	$PSD_{Mean}$	0.916	0.96
Thumb	$Amplitude_{Mean}$	0.07	0.48	Thumb	$Surface_{Freq}$	0.089	0.75
Extensor	$Amplitude_{Max}$	0.018	0.55	Thumb	$Median_{Freq}$	0.08	0.69
Extensor	$Amplitude_{Mean}$	0.042	0.54				
Extensor	$Median_{Freq}$	−0.0011	−0.47				

**Table 8:** PC coefficients and correlations between features and corresponding PC for the fifth scenario. Only |r|>0.4 are shown.

	PC1		
	90.3%		
**Muscle**	**Feature**	**Coefficient**	**Correlation**
Thumb	$Amplitude_{Mean}$	0.12	0.81
Thumb	$EMG_{Surface}$	0.68	0.98
Extensor	$Amplitude_{Max}$	0.017	0.67
Extensor	$Amplitude_{Mean}$	0.017	0.54
Extensor	$EMG_{Surface}$	−0.011	−0.42
Extensor	$Median_{Freq}$	0.04	0.72

**Table 9:** Repeated ANOVA tests over selected EMG features.

Feature	*F*-Statistic	*p*-Value
$Thumb_{Max}^{Amplitude}$	3.89	0.0124
$Thumb_{Surface}^{EMG}$	2.89	0.0403
$Extensor_{Surface}^{EMG}$	3.01	0.0348

**Table 10:** Repeated ANOVA tests over selected EEG features.

Feature	*F*-Statistic	*p*-Value
$FC5_{Max}^{\theta }$	3.12	0.0304
$P7_{Max}^{\delta }$	4.82	0.043
$AF4_{Max}^{\theta }$	5.57	0.00198
$AF4_{Max}^{\alpha }$	4.43	0.006
$AF4_{Max}^{\beta }$	3.18	0.028
$AF4_{Max}^{\gamma }$	2.844	0.0426
${\left(AF4-AF3\right)}_{Max}^{\theta }$	7.36	0.003
${\left(AF4-AF3\right)}_{Max}^{\alpha }$	6.26	0.001
${\left(AF4-AF3\right)}_{Max}^{\beta }$	3.086	0.031
${\left(AF4-AF3\right)}_{Max}^{\gamma }$	3.102	0.031
${\left(F4-F3\right)}_{Max}^{\gamma }$	2.91	0.04

## Data Availability

Data sharing is not applicable to this article.
